# COST-BENEFIT ANALYSIS OF COMMUNITY-BASED SERVICES AS A MEANS OF PROMOTING ACTIVE AGING IN GALILEE

**DOI:** 10.1093/geroni/igz038.1098

**Published:** 2019-11-08

**Authors:** Shira Hantman

**Affiliations:** Tel Hai College, Upper galilee, Israel, Israel

## Abstract

Increase in life expectancy has benefits, but also costs from increased expenses related to morbidity and prevention. These costs may be reduced by adopting a healthier lifestyle. The goal of the study was to quantify the economic value of a variety of activities in which older adults partake: e.g. cultural, intellectual, physical and nutritional activities. Research questions: Are the monetary benefits of improved health different when measured on a subjective willingness-to-pay (WTP) approach or on an objective Cost-of-Illness (COI) approach? Is the monetary benefit of active aging larger than the cost associated in doing that? 300 older adults participating in various activities of the local senior center and a control group not participating filled out a life style survey. A choice modelling (CM) approach estimated a subjective monetary welfare and compared it to an objective measure of benefit associated with the occurrence of different health symptoms associated with more active aging. An association was found between the various activities explored and the objective and subjective perspective of health. Moreover, all older adult activities passed the cost benefit test albite the order was different between objective and subjective estimations. Nutrition related activities were found to be the most beneficial. Cultural activity ranked second objectively and subjectively. Intellectual activity ranked last objectively and physical activity ranked last subjectively. Participants will understand the need to provide optimal policy and efficient resource distribution between the various activities offered to older adults. This will result in better health lowering public health expenditures.

